# Feasibility and acceptability of ExerciseGuideUK for those living with and beyond lung cancer: a mixed methods study

**DOI:** 10.1007/s00520-026-10858-w

**Published:** 2026-06-12

**Authors:** Jordan Curry, Mark Pearson, Camille E. Short, Corneel Vandelanotte, Holly E. L. Evans, Michael Lind, Cynthia C. Forbes

**Affiliations:** 1 Wolfson Palliative Care Research Centre, Hull York Medical School, University of Hull, Cottingham Road, Hull, UK; 2 The Activity and Nutrition in Cancer Research Group, Hull York Medical School, University of Hull, Kingston-Upon-Hull, UK; 3 Academic Department of Oncology, Queen’s Centre for Oncology and Haematology, Castle Hill Hospital, Cottingham, Hull UK; 4 Melbourne Centre for Behaviour Change, Melbourne School of Psychological Science, The University of Melbourne, Parkville, VIC Australia; 5 CanRex-Supporting Recovery and Exercise Research Group, Melbourne School of Health Sciences, The University of Melbourne, Parkville, VIC Australia; 6 Appleton Institute, Physical Activity Research Group, Central Queensland University, North Rockhampton, QLD Australia; 7 School of Medicine, College of Nursing and Health Sciences, Flinders University, Bedford Park, South Australia Australia; 8 iNform Research Institute, iNform Health and Fitness, Adelaide, South Australia Australia; 9 Present Address: Hull York Medical School, University of Hull, Allam Medical Building 3Rd Floor, Cottingham Road, Kingston-Upon-Hull, East Yorkshire HU6 7RX UK

**Keywords:** Physical activity, Exercise, Lung cancer, Telehealth, EHealth, Feasibility, Acceptability

## Abstract

**Purpose:**

Digital health interventions offer promise for addressing unmet needs in individuals living with and beyond lung cancer (LWBLC). ExerciseGuideUK, a personalised, web-based exercise platform, was developed for those LWBLC. This study aimed to evaluate ExerciseGuideUK.

**Methods:**

Participants were recruited from Hull University Teaching Hospital and used ExerciseGuideUK over eight weeks. Feasibility and acceptability were assessed using quantitative measures, including the System Usability Scale, structured questionnaires, and qualitative interviews (guided by the Theoretical Framework of Acceptability). A novel mixed-methods integration approach called the Pillar Integration process (PIP) was used to integrate quantitative and qualitative findings on feasibility, engagement, barriers, and adaptation potential.

**Results:**

Eighteen participants (mean age 65 ± 14.42y) enrolled. Recruitment and retention rates were 30.5% and 77%, respectively. Notably, 25.4% of those screened were excluded due to digital access issues, including some participants who owned smartphones, suggesting limited digital literacy. Among those who participated, acceptability, usability, and engagement were high. Valued features included goal-setting tools, breathlessness management strategies, and personalised exercise prescriptions. Barriers included treatment side effects, work responsibilities, and low confidence with technology.

**Conclusion:**

ExerciseGuideUK showed moderate feasibility and high acceptability among users. This is the first known application of PIP in the context of lung cancer, exercise, and digital health. The PIP facilitated a comprehensive understanding of feasibility and acceptability, guiding informed adaptations by users. The PIP warrants further testing and refinement in relation to optimising digital intervention design that promotes equitable, patient-centred care.

**Trial registration:**

ClinicalTrials.gov Identifier: NCT05121259.

**Supplementary Information:**

The online version contains supplementary material available at 10.1007/s00520-026-10858-w.

## Introduction

Lung cancer remains one of the most prevalent and deadly cancers worldwide [1]. Advances in early detection and treatment have improved survival rates, meaning more individuals living with and beyond lung cancer (LWBLC) [2]. However, this population faces many challenges, including physical deconditioning, fatigue, breathlessness, and psychological distress, which can severely impact quality of life (QoL) [3]. Compared with other cancers, lung cancer has been identified as having one of the highest unmet symptom burdens [4, 5]. Lung cancer patients tend to be elderly and have multiple comorbidities, making therapeutic interventions challenging [6].

Exercise interventions offer promise in addressing these challenges, with evidence highlighting improvements in physical function, psychological well-being, and overall QoL for those LWBLC [7, 8] and the larger cancer population [9]. Despite these benefits, this population's participation in structured exercise programmes remains limited, possibly due to barriers like physical health limitations, insufficiently tailored programmes, psychological factors, and accessibility challenges [10]. In England, geographic disparities and resource constraints are a concern for the accessibility of services [11].

Digital health interventions offer promise in overcoming these barriers. Recent reviews [12, 13] looking at eHealth interventions among people LWBLC show high feasibility and acceptability, and positive trends in better physical function and QoL. However, most included studies were small feasibility or pilot studies among surgical populations, and none were evaluated as routine clinical practice [12]. This population's age, comorbidity burden, lower fitness, and digital literacy present real challenges to uptake and engagement [14–16] that existing platforms have rarely addressed with lung cancer-specific content.

To address these gaps, ExerciseGuideUK, a personalised, web-based exercise platform, adapted from an existing platform [17], was specifically developed for individuals LWBLC. Distinct from prior work, it incorporates lung cancer-specific content, breathlessness management and progressive exercise prescription, across disease stages and treatment types, within a flexible, home-based format. This programme integrates evidence-based exercise recommendations (e.g., NHS and ACSM) to ensure safety, adaptability, and patient-centred care. Delivered through a flexible, home-based format with options for gym or community use, ExerciseGuideUK aimed to enhance access and engagement, encouraging individuals to incorporate physical activity (PA) and healthy lifestyle behaviours into their daily lives despite the barriers they may face.

This study aimed to evaluate the feasibility and acceptability of ExerciseGuideUK using a mixed-methods approach. To synthesise qualitative and quantitative data, the Pillar Integration Process (PIP) [18] was employed. This is a structured, four-stage technique designed to systematically and reproducibly integrate different data sources. Using the PIP provided a comprehensive and nuanced understanding of the unique needs of those LWBLC and informs future digital health interventions.

## Methods

Full details of the development and adaptation of ExerciseGuideUK from prior iterations, recruitment and data collection methods, patient and clinician involvement, intervention content and delivery methods, and application of the Behaviour Change Technique (BCT) Taxonomy are described in the published study protocol [19] and a public involvement process commentary [20]. Public involvement can be summarised as research conducted 'with' or 'by' members of the public, rather than 'to', 'about' or 'for' them [21]. The following methods section will briefly summarise this content. A full breakdown of BCTs applied across each website component is also provided in Supplement 5.

### Study design & participants

This was a single-arm, non-randomized, feasibility study. Clinicians identified eligible participants (criteria in Table 1) at a large, single NHS site in Northern England, during routine visits and, if interested, referred them to the researcher; reasons for ineligibility or refusal were recorded if provided. Participant flow is presented in the protocol paper [19]. We aimed to recruit 15—35 individuals LWBLC. This sample size was informed by clinical and academic expertise, relevant literature on online supportive care for those LWBLC, an audit of sample sizes for feasibility trials in the UK [13, 22], and would be feasible within a doctoral degree. See protocol paper for details [19].

**Table 1:** Eligibility criteria

Inclusion	Exclusion
Be 18 years or older	Access to a laptop, computer, or smart device to aces the web-based platform
Have had a lung cancer diagnosis	Cogitative or linguistically inability to provide informed consent
Be able to speak and read in English	Physical or cognitive imperilments which prevent or inhibit participation in physical activity
Willing and able to provide informed consent	Bone metastasis in weight-bearing locations and/or spinal compressions
Have internet connectivity	
Access to a laptop, computer, or smart device to access the web-based platform	

### Intervention & data collection

Participants received a standalone eight-week personalised exercise prescription and additional health-behaviour resources via ExerciseGuideUK. Eight weeks was selected based on the duration of the original ExerciseGuide platform [17], precedent from digital exercise interventions in lung cancer [12], and to support the early stages of habit formation and consolidation, with behavioural automaticity beginning to develop within this timeframe [23].

ExerciseGuideUK led participants sequentially through educational modules and automatically recorded engagement (via Google Analytics) [19]. At first login, participants first completed the baseline questionnaire, then were guided through a platform orientation, including how to navigate key features such as buttons, videos, and dropdown menus, and an overview of how the programme worked.

Exercise prescription focused on aerobic and resistance training, supplemented by balance and flexibility activities, aligned with NHS and ACSM guidelines, and included dedicated progression and exercise tracking in the Tracking module. Exercise prescriptions were generated from ExerciseGuideUK surveys, using *IF–THEN* coding algorithms. For full details, see protocol [19]. Behaviour change support was embedded in modules and reminders throughout, incorporating techniques from BCT Taxonomy [24], such as goal-setting, action-planning, and self-monitoring with automated feedback within the modules (feedback updated every time a module was edited by participants). See Supplement 5 for more detail. Full details of the intervention delivery and components including a breakdown of each module, its tailoring process and BCTs is provided in the protocol paper [19]. ExerciseGuideUK generated the personalised prescriptions, adapted if needed by the researcher, at both baseline and a mid-programme review. Therefore, every participant had at least two contacts with the researcher, alongside the web-based content. Post-study interviews and surveys were conducted online within two weeks of finishing the programme. 

### Feasibility

Feasibility was evaluated using recruitment and retention rates. Recruitment feasibility was determined by achieving a pre-defined target rate of ≥ 60%. Retention was assessed as the proportion of participants completing the eight-week study, with a target retention rate of ≥ 85%.

Descriptive statistics (e.g., means, standard deviations, percentages) were used to summarise the recruitment rate (proportion of eligible participants who provided consent), retention data, and explore dropouts.

Qualitative feedback was gathered through online (via Zoom) post-study interviews and open-ended end-of-study questionnaire items, focusing on barriers, motivators, satisfaction, and usability influencing recruitment and retention. Open-ended responses were analysed descriptively and integrated into the mixed-methods PIP alongside interview data. End-of-study questionnaires also included Likert-scale items; results are presented in the acceptability findings. Full details of questionnaire items are provided in the study protocol [19].

### Acceptability

Acceptability was evaluated through post-study interviews guided by the Theoretical Framework of Acceptability (TFA) [25] and the System Usability Score (SUS), with scores above 68 indicating acceptable usability [26]. Each participant’s scores were graded [27, 28] and summarised descriptively to evaluate user satisfaction.

### Secondary outcomes

Secondary outcomes included: 1) patient-reported outcomes (PROs; activity levels, health-related quality of life, depression, and anxiety), 2) website engagement, and 3) safety, detailed in the study protocol [19]. PRO data were captured at baseline (week 0) and post-study (week 9) through the ExerciseGuideUK platform. Engagement was quantified using Google Analytics 4 (page views, session duration, and time on page) and supported by participant comments that clarified usage patterns and highlighted barriers. Safety was examined both quantitatively, by reported adverse events, and qualitatively, by post-study interviews.

### Data analysis & mixed methods integration

Data were analysed in SPSS (IBM, Version: 29, 2023). Descriptive statistics (means(SD) or medians, inter-quartile range (IQR)) summarised secondary outcomes (EORTC-QLQ-C30, HADS, CHAMPS) at baseline and follow-up. Change scores were explored and generated from ~ 10,000 bootstrap resamples due to a small target sample. Participants with data at both time points formed the completer sample.

All post-study interviews were transcribed verbatim and managed in NVivo. A deductive-inductive thematic analysis was undertaken [29]. Two researchers (JC and MP) independently coded at least 25% of the transcripts (~ 29%, exceeding the 25% dual-coding benchmark for reliability [30–32]) and then met to discuss any differences. The remaining transcripts were coded by one researcher (JC) with ongoing mentor debriefing. See supplement 2 for COREQ.

For a holistic understanding of feasibility and acceptability, quantitative and qualitative data were integrated using the PIP [18]. Using the PIP approach, a separate Microsoft Word table was set up for both feasibility and acceptability, with the four PIP stages applied within every table. Stages include: 1) Listing: raw quantitative results (e.g., consent, dropout reasons, survey scores) were placed in the left-hand column, while relevant qualitative excerpts were placed in the right column, keeping the two data types separate but visible, 2) Matching: related rows were grouped into shaded blocks and aligned horizontally. Where no match was possible, the cell was tagged “Not reported”, mirroring the table, 3) Checking: Two reviewers (JC, MP, or CF) revisited raw data to confirm completeness and agreement, resolving any discrepancies and ensuring each quote accurately reflected its adjacent data point, and 4) Pillar Building: verified blocks were collapsed into broader themes, also known as "Pillars". Table 2 provides definitions of the stages and their application.

**Table 2:** The key stages of the Pillar Integration Process, their definition [18], and the application to the ExerciseGuideUK study

Stage	Definition	Application
Listing	This stage involves organising raw data from quantitative and qualitative analyses into categories or codes deemed relevant for integration. These data points, such as percentages, coded themes, or selected quotations, are listed separately to preserve their distinct nature while setting the foundation for cross-comparison	Multiple datasets were combined, including consent data, dropout information, reasons for declining participation, survey data, and post-study interviews. Data was mapped to provide a holistic overview, starting with quantitative data
Matching	Data listed from one type (e.g., quantitative) are aligned horizontally with data from the other type (e.g., qualitative) to identify patterns, relationships, or gaps. This alignment involves refining categories, recognising parallels, and highlighting convergences or divergences in the datasets	Numerical trends with interview narratives were aligned, using thematic analysis guided by the Theoretical Framework of Acceptability to identify patterns. Instances where qualitative or quantitative data couldn’t be matched were labeled as “Not reported.”
Checking	Once the data are matched, they are cross-checked for quality and completeness to ensure all rows in the display are appropriately aligned. Any identified gaps are re-examined against the raw data to confirm patterns or the absence of data, refining and validating the integration process	Raw data was revisited to confirm that all relevant data were captured. Although no significant gaps or inconsistencies were identified, the review of raw data ensured the integrity of the matching process. This step reinforced the coherence of the integration
Pillar Building	The final stage synthesises matched data into overarching themes or “pillars” that encapsulate the insights gained from integrating quantitative and qualitative findings. These pillars represent unified narratives or conclusions derived from the combined datasets	Thematic pillars were established regarding feasibility (n = 2) and acceptability (n = 7). By grouping matched data, integrative pillars were created, representing insights across quantitative and qualitative paradigms. Tables were used to visually organise these pillars

## Results

### Participants

A convenience sample of 130 patients LWBLC were screened during routine visits to the oncology clinics. Patients were approached in person by the researcher and assessed for eligibility.

**Fig. 1:**
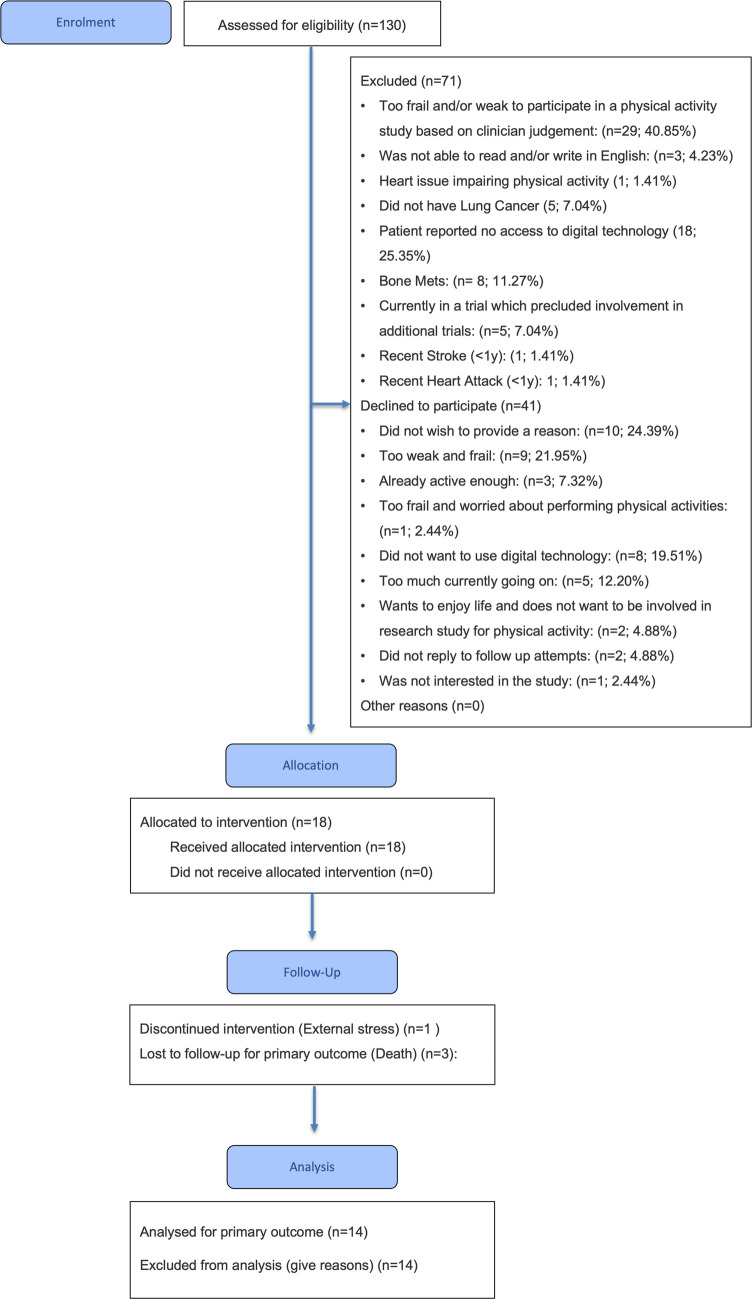
Modified Consolidated Standards of Reporting Trials (CONSORT) diagram for a single-arm, nonrandomised, feasibility study

See modified CONSORT diagram in Fig. 1.

Participant characteristics are presented in Table 3.

**Table 3:** Participant Characteristics

	Total (*n* = 18)	Completed (*n* = 14)^†^
Age	65 (± 14.42)	68.43 (± 9.20)
Height (cm)	172.24 (± 10.58)	174.29 (± 10.21)
Weight (kg)	86 (± 23.57)	84.79 (± 26.58)
Sex		
Male	10 (55.6%)	9 (63.3%)
Female	8 (44.4%)	5 (35.7%)
Ethnicity		
White British	18 (100%)	14 (100%)
Relationship Status		
Married/Common Law	12 (66.7%)	10 (71.4%)
In a relationship (not living together)	1 (5.6%)	1 (7.1%)
Single/Never married	1 (5.6%)	1 (7.1%)
Divorced/Separated	2 (11.1%)	1 (7.1%)
Widowed	2 (11.1%)	1 (7.1%)
Employment		
Employed (Full Time)	3 (16.7%)	3 (21.4)
Employed (Part Time)	1 (5.6%)	0 (0%)
Employed (Casually)	0 (0%)	0 (0%)
Currently Unemployed	0 (0%)	0 (0%)
Retired	14 (77.8%)	11 (78.6)
Student	0 (0%)	0 (0%)
Homemaker	0 (0%)	0 (0%)
Currently on Disability	0 (0%)	0 (0%)
Cancer Stage		
Stage I	0 (0%)	0 (0%)
Stage II	2 (11.1%)	2 (14.3%)
Stage III	2 (11.1%)	1 (7.1%)
Stage IV	3 (16.7%)	3 (21.4%)
Do not know	11 (61.1%)	8 (57.1%)
Currently Receiving Treatment		
Yes	15 (83.3%)	12 (85.7%)
Current Treatments		
Chemotherapy	5 (27.8%)	4 (28.6%)
Immunotherapy	8 (44.4%)	7 (50%)
Surgery	7 (38.9%)	5 (35.7%)
Targeted Drug Treatment	5 (27.8%)	4 (28.6%)
Radiotherapy	6 (33.3%)	4 (28.6%)
Previous Treatments		
Chemotherapy	7 (38.9%)	6 (42.9%)
Immunotherapy	6 (33.3%)	5 (35.7%)
Surgery	8 (44.4%)	6 (42.9%)
Targeted Drug Treatment	3 (16.7%)	3 (21.4%)
Radiotherapy	6 (33.3%)	5 (35.7%
Comorbidities		
Lung Disease	15 (83.3%)	12 (85.7%)
Diabetes	11 (61.1%)	7 (50%)
High Blood Pressure	13 (72.2%)	10 (71.4%)
High Blood Cholesterol	12 (66.7%)	10 (71.4%)
Arthritis	15 (83.3%)	11 (78.6%
Stroke	8 (44.4%)	7 (50%)
Other Cancers	11 (61.1%)	9 (64.3%)
Angina	9 (50%)	7 (50%)
Depression	10 (55.6%)	7 (50%)
Lymphedema	8 (44.4%)	7 (50%)
Poor circulation to legs or feet	11 (61.1%)	8 (57.1%)
Smoking Status		
Never Smoker	4 (22.2%)	4 (28.6%)
Occasional Smoker	1 (5.6%)	1 (7.1%)
Ex-Smoker	13 (72.2%)	9 (64.3%)
Self-Reported Overall Health Rating		
Poor	2 (11.1%)	1 (7.1%)
Fair	8 (44.4%)	5 (35.7%)
Good	6 (33.3%)	6 (42.9%)
Very Good	2 (11.1%)	2 (14.3%)

Data are presented in *n* (%) or mean (SD)

^†^One withdrew and three died

### Mixed methods integration

**Fig. 2:**
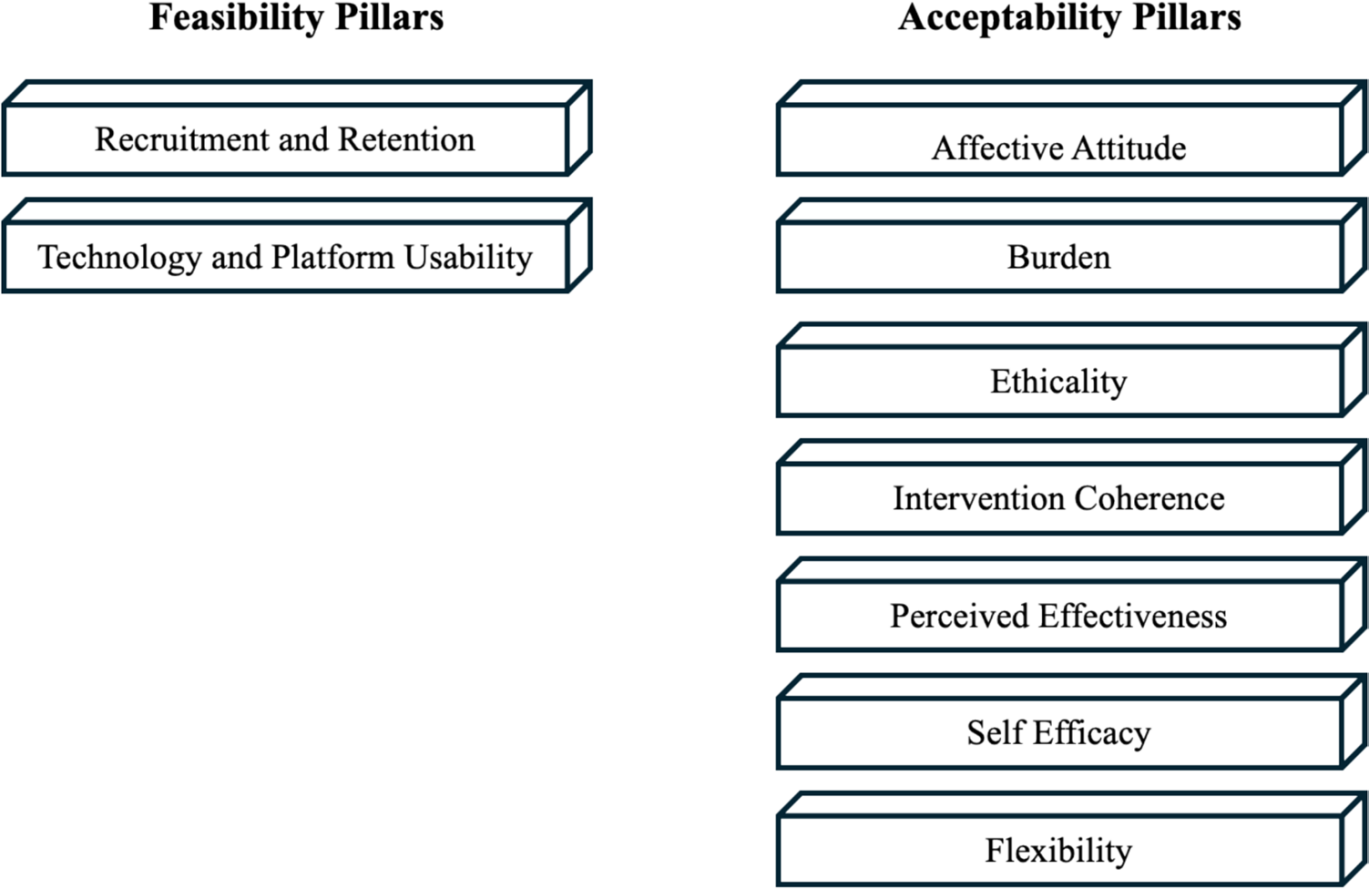
Illustrating the Mixed-Methods process using the Pillars from the Pillar Integration Process. Acceptability Pillars are structured around the Theoretical Framework of Acceptability [25]

The main findings answer two questions: 1) Is ExerciseGuideUK feasible for those LWBLC? 2) Is ExerciseGuideUK acceptable for those LWBLC? For each question, quantitative data and interview excerpts were integrated into distinct pillars using the PIP, comprising two feasibility and seven acceptability pillars (Fig. 2). Each subsection below provides results highlighting where the data types converge, diverge, or provide complementary context. Some secondary outcome data is detailed throughout the results section where it supports the overall pillars. For additional secondary outcome results, we have included pre-post analysis data for self-reported activity, health-related quality of life, anxiety, and depression in Supplement 6.

### ExerciseGuideUK feasibility

#### Feasibility pillar one: recruitment and retention

The recruitment rate was 30.5%, below the predefined target of ≥ 60%. Retention was 77%, lower than ≥ 85% target. The top three reasons for declining participation were 1) no reason provided (24.39%), 2) being too weak/frail (21.95%), and 3) reluctance to engage with digital technology (19.51%). Interviews provided further insight into reduced participation and attrition, with treatment side effects and fatigue as primary barriers:

*“Three days after chemo, I was completely knocked out. I couldn’t even think about logging on.” (P008, 74M).*

Despite this, 12 out of 18 (66.67%) participants completed full PRO measures, suggesting good compliance among retained participants.

#### Feasibility pillar two: technology and platform usability

When approached, ~ 25% of potential participants reported no access to digital technology, and approximately 20% declined participation due to digital discomfort.

While some participants rated usability favourably:

*“I found no grave problems. It was easy to use.” (P005, 77M)*

Others described difficulties with digital literacy and navigation:

*“Older people like me would find this daunting…it would be easier with a printed guide.” (P002, 75M).*

ExerciseGuideUK was positively received, with SUS results of excellent (n = 2), good (n = 6), and poor/awful (n = 4) ratings, and an overall score of 72%.

Together, the positive usability comments and higher SUS ratings converge on usability, while recommendations of paper-based guides and the widespread SUS scores reveal divergence in participant experience. See Supplement 3 for further data.

### ExerciseguideUK acceptability

**Fig. 3:**
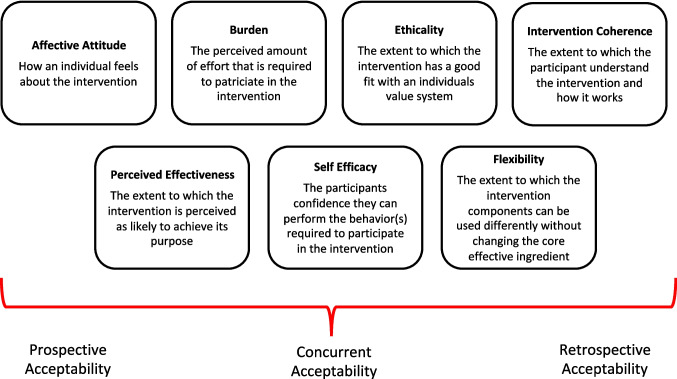
A modified Theoretical Framework of Acceptability (v2), authored by Sekhon et al. (2017), of the constructs used within the analysis of the acceptability of ExerciseGuideUK [25]

Acceptability was examined across the seven TFA domains, integrating usability scores with participant perspectives. The TFA was modified inductively (guided by the dataset), resulting in the Opportunity Cost construct being replaced by Flexibility. See Fig. 3 for modified TFA, and further data in Supplement 4.

#### Acceptability pillar one: affective attitude – “how do I feel about it?”

Participants expressed enjoyment and motivation for behaviour change:

*“I actually look forward to doing my exercises… I’ve enjoyed, you know doing the exercises.” (P013, 70M).*

However, some either required external motivation or advocated the importance of motivation from others:

*“If my wife wasn’t still behind me, I don’t think I would have done it [exercises].” (P008, 74M).*

*“I think it's [ExerciseGuideUK] very, very good. And I think the main thing that you and your colleagues have got to possibly get through to people is to try and get them interested” (P005, 77M).*

Yet, when answering if the ‘website added value to my cancer care and service’, the rating was high (M(SD) = 4.08(0.86)).

Participants felt the website was well designed:

*“Well, me personally, I just thought. I thought it was set out well. I thought there was lots of explanatory things.” (P014, 71 F)*

Which is supported by good scores for professionalism, appropriate content, colour, and images (M(SD) = 4.15(0.69)).

Interviews highlighted intrinsic enjoyment and reliance on social/family encouragement. Yet, these divergent drivers converge in overall positive affect, reflected by high mean ratings for added value and presentation quality.

#### Acceptability pillar two: burden – “how hard is this to fit in?”

Although the website’s perceived burden was low (M(SD) = 1.62(0.65)), it elicited some negative emotions (M(SD) = 1.54(0.78)). Participants’ overall high burden from treatment side effects and work commitments meant that even modest demands could feel onerous:

*“I work 12-hour shifts, and I’m exhausted when I get home.” (P003, 51 F).*

*“So, one of the side effects for me of the medication, you know, it's an effect of my eyesight. So, you know, reading things has became a bit of a chore… I've struggled to concentrate on some of the writing.” (P012, 54 F)*

Moreover, navigation appeared straightforward:

*“…funny enough, I wouldn't consider myself computer illiterate, but I found this very easy to use.” (P005, 77M)*

*“Yes, yes. And the diagrams, yes.… Like I said, I've not found any difficulty with it at all.” (P014, 71y)*

Taken together, quantitative ratings and usability scores suggest low intrinsic burden for the website, whereas interview excerpts concurrently document clear treatment- and work-related demands.

#### Acceptability pillar three: ethicality – “does this line up with what matters to me?”

ExerciseGuideUK was widely regarded as aligned with personal values and long-term health goals.

*“I knew I was getting worse, and I knew I would definitely have gotten worse if I didn’t do some exercise. I know the benefits.” (P004, 84M).*

*“Even before you introduce me to the study, well into the study I had searched out various websites” (P017, 64M)*

Interview data indicated high ethicality; participants viewed ExerciseGuideUK as fitting with their values and health goals, supporting efforts to stay active, limit decline, and seek trusted exercise information.

#### Acceptability pillar four: flexibility – “can I use it my way?”

Participants valued the website’s flexibility; its exercise content and tracking tools could be accessed in multiple ways, with some users opting for printed materials rather than digital formats.

*“I like to keep it on paper. I like to see things written down.” (P013, 70M).*

*“And I thought you know what? Me and him talk anyway, so I'll just tell him how well I'm doing. But yeah, my phone and watch. Have been my personal way of tracking, I suppose.” (P012, 54 F)*

Interview data converged on high flexibility. Participants selected the modality (e.g., paper logs, website, phone/watch apps) that best suited their routines, indicating the intervention could be personalised to delivery or tracking preferences.

#### Acceptability pillar five: intervention coherence – “do I get how this works?”

Goal-setting and exercise progression were strong motivational factors, with participants emphasising the importance of steadily increasing difficulty:

*“Yeah, you know, it [exercise intensity] went up. They went up as the weeks went along, didn't it? You know, the amount of reps of the actual exercises went up, so I thought that was good.” (P003, 51 F)*

*“You know, I'm on week nine now. I'm hoping in a couple of weeks' time, instead of doing 12 repetitions, I'm hoping I can take up to 15. In that kind of things, and that's in the future…” P013, 70M)*

*“If you don’t have a goal, you will just think, why bother? You can go down a slippery slope.” (P004, 84M).*

Staggered release of modules was perceived positively:

*“If all the modules were there at the start, I would have done them all then forgotten everything.” (P003, 51 F).*

Participants understood the programme’s logic, progressive increases in repetitions and goal-setting maintained motivation, while staggered release of modules prevented massed-learning and supported ongoing engagement.

#### Acceptability pillar six: perceived effectiveness – “is it doing anything for me?”

ExerciseGuideUK was rated highly for helping patients identify PA goals (M(SD) = 4.38(0.65)) and changing attitudes toward exercise (M(SD) = 3.77(0.93)).

Participants reported noticeable physical and psychological benefits and habit formation:

*“I physically couldn’t do it before because I couldn’t breathe properly… But now I can!” (P006, 62 F).*

*“Whereas now. I would find it difficult not to do it if that would make sense…It [exercising] becomes very much part of your day. And your daily routine.” (P002, 75M)*

Additionally, participants highlighted improvements in breathlessness management, suggesting the intervention contributed to functional gains:

*“One of the biggest things I have learned is that I’m not afraid of being breathless anymore.” (P013, 70M).*

Ratings showed high perceived effectiveness for both goal identification and attitude change toward exercise. Consistent with scores, participants reported physical benefits, incorporating exercise into daily routines, and greater confidence managing breathlessness.

#### Acceptability pillar seven: self-efficacy – “can I keep doing this safely?”

Over time, participants gained confidence in exercise participation (M(SD) = 4.08(0.86)) and reported overcoming initial concerns about safety and ability:

*“At first, I thought this was a big mistake. But now I can just get on the floor and do them.” (P011, 76M).*

*"I'll be honest with you, because of the breathlessness. I, I was shying away from the cardio element because I wasn't comfortable. But now I found that I can get to a point where I can raise my heart rate for a prolonged period of time using the breathing”. (P001, 64M)*

Self-efficacy for reported exercise was supported by ExerciseGuideUK, with interviewees describing moving from initial caution to confidently performing both floor and cardio exercises. No adverse events were reported throughout the programme, supporting the safety of ExerciseGuideUK in this clinically complex population.

## Discussion

This study applied the PIP to synthesise mixed data sources, offering a holistic understanding of the feasibility and acceptability of ExerciseGuideUK. Findings indicate that while ExerciseGuideUK was well-received and perceived as beneficial, feasibility was limited by recruitment and retention challenges, digital literacy, and individual health-related constraints. By integrating quantitative usability data with qualitative participant perspectives, this study evaluated engagement patterns, participation barriers, and intervention flexibility, contributing to broader discourse on digital health interventions for individuals LWBLC.

Low recruitment and retention rates impacted feasibility. Although ExerciseGuideUK was designed to be accessible, recruitment challenges aligned with existing research demonstrate the difficulty of engaging those LWBLC in digital health interventions [13, 33, 34]. Non-eligibility was primarily attributed to clinician-determined limited ability or frailty. This reflects a broader trend where clinicians don’t approach patients for exercise studies due to concerns about capacity to tolerate PA, despite growing evidence supporting the benefits and international guidelines promoting exercise integration into cancer care [35]. Non-participation was mostly reluctance to engage with digital platforms and symptom burden, particularly fatigue and physical deterioration. Retention was primarily affected by three deaths throughout the study, with only one participant actively withdrawing. Still, attrition was influenced by progressive illness and treatment side effects, consistent with research suggesting symptom burden remains a primary barrier to long-term engagement in lung cancer-related exercise interventions [15, 36]. This supports the need for recruitment strategies that proactively address engagement barriers and reduce clinician gatekeeping. Future studies could combine routine clinician referral with self-referral options (e.g., QR codes, web sign-up) and automated prompts in electronic patient records to ensure all eligible patients are offered the programme. Embedding structured support, like computerised reminders and brief health-professional check-ins, could help sustain adherence.

Digital literacy and accessibility also impacted feasibility, with ~ 25% of participants reporting no access to digital technology and about 20% declining participation due to not wanting to use digital devices. Notably, some who owned smartphones still perceived themselves as lacking access, highlighting a gap in understanding of digital devices. While some participants found the platform straightforward, others struggled with navigation or preferred paper-based alternatives. This aligns with previous research on digital exclusion, particularly among older adults or those with limited prior exposure to online platforms [16]. In the UK, technology use among the ageing population has increased, with 67% of those 65 + using smartphones and 80% using the internet at least monthly; however, 18% still lack internet access or use [37]. Given this, future digital health interventions are recommended to adopt hybrid delivery models combining digital tools with non-digital components (e.g., paper-based plans and in-person support) to maximise accessibility and engagement among older adults [38].

Despite feasibility challenges, ExerciseGuideUK was well-accepted by participants, with high engagement across multiple TFA constructs. Participants valued the structured nature of the programme, goal-setting features, and perceived physical and psychological benefits, all of which support adherence to cancer-related exercise interventions [39–42]. Emotional engagement was a key facilitator of participation, with some participants expressing intrinsic motivation while others required external encouragement from family or researcher check-ins. This suggests embedding peer-support networks or social accountability features may enhance adherence by fostering a sense of togetherness or accountability.

While usability ratings indicated the website was not burdensome, qualitative insights highlighted treatment-related fatigue and work commitments affected participation. Participants undergoing active treatment described periods where engagement felt impossible, consistent with previous findings that fluctuating energy levels impact PA adherence in cancer populations [43]. Full-time workers struggled to integrate exercise into routines, reinforcing the need for brief, adaptable programmes. Evidence suggests shorter, more flexible exercise sessions may enhance adherence, particularly for clinical populations managing multiple competing demands [44]. Future studies should incorporate greater flexibility, allowing participants to modify exercise intensity according to work commitments and personal circumstances, engage asynchronously, and temporarily pause or suspend the programme (e.g., when sick).

Perceived effectiveness was strongly endorsed; participants reported improvements in strength, breathlessness management, and confidence in their abilities. Quantitative findings indicated high scores for goal-setting support and changes in exercise attitudes, while qualitative and quantitative data showed that ExerciseGuideUK led to physical and psychological benefits. These results align with previous studies demonstrating structured exercise interventions can enhance exercise self-efficacy and improve symptom management in individuals LWBLC [45, 46]. Breathlessness management was a particularly meaningful outcome; many participants initially expressed uncertainty about whether they could exercise safely. However, over time, participants reported greater confidence in their ability to regulate breathing, reinforcing the value of structured guidance in alleviating concerns around PA, showing that even a simple intervention can reduce barriers and encourage activity. Since breathlessness is a major barrier to exercise for individuals LWBLC [14], integrating breathlessness-specific modules and guided exercises earlier in interventions (e.g., breathing techniques) could improve impact.

### Strengths and limitations

Using the PIP, this study bridged quantitative recruitment, retention, and system usability measures with rich qualitative narratives about participant experiences. This integration not only deepened the interpretation of findings but also facilitated actionable insights for refining ExerciseGuideUK, ensuring that it effectively meets the needs of individuals LWBLC. The sample size (n = 18) aligns with feasibility recommendations suggested by Billingham et al., [22], yet the brief recruitment period and limited diversity, particularly a younger cohort compared to the UK average age of lung cancer diagnosis between 85–89 years [47], limit generalisability. Digital inequity further constrained participation, impacting overall feasibility. The sample was exclusively White British, limiting generalisability to ethnically diverse populations who may face additional or distinct barriers to digital health engagement. Recruitment from a single centre further constrains geographic generalisability. Additionally, the majority of participants were undergoing active treatment (83.3%), meaning those in survivorship, not receiving treatment, are underrepresented. Despite these limitations, the study provides valuable insight into ExerciseGuideUK’s acceptability and offers a strong foundation for refining digital health interventions for individuals LWBLC.

## Conclusion

Barriers to engagement centred around health-related factors, digital literacy, work commitments, and concerns over exercise safety. Treatment-related fatigue and progressive illness were key barriers to participation, contributing to both non-engagement and attrition. However, for those retained, ExerciseGuideUK was perceived as a valuable tool for symptom management and rehabilitation, with self-efficacy increasing over time, reinforcing the potential role of structured digital interventions in cancer care [48, 49].

The current findings suggest moderate feasibility and high acceptability, providing early support for progression toward a larger feasibility trial with targeted amendments.

Whilst pre-specified targets were not met, recruitment took place during the COVID-19 pandemic (2022/2023), when strict clinical restrictions substantially limited researcher access to eligible patients, and most attrition was attributable to participant deaths rather than disengagement.

Revised recruitment strategies, hybrid delivery models to accommodate digital literacy barriers, and flexible engagement options during active treatment are recommended adaptations prior to conducting a larger trial.

## Supplementary Information

Below is the link to the electronic supplementary material.

Supplementary file1 (DOCX 20 kb)

Supplementary file2 (DOCX 24 kb)

Supplementary file3 (DOCX 42 kb)

Supplementary file4 (DOCX 25 kb)

Supplementary file5 (DOCX 29 kb)

Supplementary file6 (DOCX 70 kb)

## Data Availability

No datasets were generated or analysed during the current study.
