# Supplementary material for: Feasibility and acceptability of ExerciseGuideUK for those living with and beyond lung cancer: a mixed methods study
Source: Support Care Cancer. 2026 Jun 12;34(7):646. doi: 10.1007/s00520-026-10858-w (PMC13260022; doi:10.1007/s00520-026-10858-w)
Supplement: Supplementary file 1 — Supplementary file1 (DOCX 20 kb) [file 520_2026_10858_MOESM1_ESM.docx]

Supplement 2: CONSOLIDATED CRITERIA FOR REPORTING QUALITATIVE RESEARCH (COREQ): 32-item checklist

| **No.** | **Item** | **Guide Questions/Description** | **Information** |
| --- | --- | --- | --- |
| Domain 1: Research team and reflexivity  Personal characteristics | | | |
| 1 | Interviewer/facilitator | Which author/s conducted the interview or focus group? | JC conducted the interviews |
| 2 | Credentials | What were the researcher’s credentials? E.g. PhD, MD | Bachelor’s of Science and Masters of Science |
| 3 | Occupation | What was their occupation at the time of the study? | Doctoral Postgraduate Research Student |
| 4 | Gender | Was the researcher male or female? | Male |
| 5 | Experience and training | What experience or training did the researcher have? | JC held a Postgraduate Certificate in Research Practice, which contained qualitative and quantitative research methods and interview training. |
| Relationship with participants | | | |
| 6 | Relationship established | Was a relationship established prior to study commencement? | The researcher was the participants trainer in throughout the study. This included check-ins. |
| 7 | Participant knowledge of the interviewer | What did the participants know about the researcher? e.g., personal goals, reasons for doing the research | Participants knew the reasons for doing the research and their professional background. |
| 8 | Interviewer characteristics | What characteristics were reported about the inter viewer/facilitator? e.g., Bias, assumptions, reasons, and interests in the research topic | Not Reported |
| Domain 2: Study design  Theoretical framework | | | |
| 9 | Methodological orientation and Theory | What methodological orientation was stated to underpin the study? e.g., grounded theory, discourse analysis, ethnography, phenomenology, content analysis | A mixed-methods study with thematic analysis being used. |
| Participant selection | | | |
| 10 | Sampling | How were participants selected? e.g., purposive, convenience, consecutive, snowball | Convenience sample |
| 11 | Method of approach | How were participants approached? e.g., face-to-face, telephone, mail, email | Face-to-face in hospital clinic |
| 12 | Sample size | How many participants were in the study? | 18 allocated to intervention |
| 13 | Non-participation | How many people refused to participate or dropped out? Reasons? | Four. One external stress and three deaths |
| Setting |  | | |
| 14 | Setting of data collection | Where was the data collected? e.g., home, clinic, workplace | Online |
| 15 | Presence of nonparticipants | Was anyone else present besides the participants and researchers? | No |
| 16 | Description of sample | What are the important characteristics of the sample? e.g., demographic data, date | This is reported in Table 2. |
| Data collection | | | |
| 17 | Interview guide | Were questions, prompts, guides provided by the authors? Was it pilot tested? | A topic guide was used and discussed with a Patient and Public Involvement group |
| 18 | Repeat interviews | Were repeat interviews carried out? If yes, how many? | No |
| 19 | Audio/visual recording | Did the research use audio or visual recording to collect the data? | Audio |
| 20 | Field notes | Were field notes made during and/or after the interview or focus group? | No |
| 21 | Duration | What was the duration of the inter views or focus group? | Interviews ranged from 30 – 90 minutes |
| 22 | Data saturation | Was data saturation discussed? | No |
| 23 | Transcripts returned | Were transcripts returned to participants for comment and/or correction? | No |
| Domain 3: analysis and findings  Data analysis | | | |
| 24 | Number of data coders | How many data coders coded the data? | Two |
| 25 | Description of the coding tree | Did authors provide a description of the coding tree? | Coding is discussed in Methods and Results |
| 26 | Derivation of themes | Were themes identified in advance or derived from the data? | Inductive and Deductive approach used |
| 27 | Software | What software, if applicable, was used to manage the data? | NVIVO and SPSS |
| 28 | Participant checking | Did participants provide feedback on the findings? | No |
| Reporting | | | |
| 29 | Quotations presented | Were participant quotations presented to illustrate the themes/findings? Was each quotation identified? e.g., participant number | Yes |
| 30 | Data and findings consistent | Was there consistency between the data presented and the findings? | Yes |
| 31 | Clarity of major themes | Were major themes clearly presented in the findings? | Yes |
| 32 | Clarity of minor themes | Is there a description of diverse cases or discussion of minor themes? | See the Results and Discussion. |
| Notes: | | | |
| **No.** | **Item** | **Guide Questions/Description** | **Reported on Page No.** |
