# Supplementary material for: Feasibility and acceptability of ExerciseGuideUK for those living with and beyond lung cancer: a mixed methods study
Source: Support Care Cancer. 2026 Jun 12;34(7):646. doi: 10.1007/s00520-026-10858-w (PMC13260022; doi:10.1007/s00520-026-10858-w)
Supplement: Supplementary file 2 — Supplementary file2 (DOCX 24 kb) [file 520_2026_10858_MOESM2_ESM.docx]

Supplement 3: Pillar Integration Process Table discussing whether the ExerciseGuide UK platform was feasible for those Living with and Beyond Lung Cancer.

| **Quantitative Findings** | **Quantitative Categories** | **Pillar Building Themes** | **Qualitative Categories** | **Qualitative Findings** |
| --- | --- | --- | --- | --- |
|  | |  |  | |
| Recruitment Rate: 30.5% | Recruitment and Retention | Recruitment and Retention | Participation | **P:** Yes, yes, you asking me because, you know, you can have leaflets and things like that. But some people wouldn't even bother reading them, would they? But yeah, it was you saying, 'would you mind getting involved (005, 77y) |
|  |  |  |  | I think because it was the chance to do something positive. And something, you know, this is something I'm not going to recover from. I think an exercise programme like this. I felt as if I was doing something positive. And this it's good for you mentally as well, isn't it? (002, 75y) |
| Retention Rate: 77% |  |  |  | R…was there any times where you didn't really want to use the website or exercise?  **P:** Yes. Particularly around three days after the chemo, you know? That really knocked me out for about three days…I just got knocked out (008, 74y) |
| Retention Challenges Due to Health-Related Issues (three participants lost due to death) |  |  |  | **P:** So, one of the side effects for me of the medication, you know, it's an effect of my eyesight. So, you know, reading things has became a bit of a chore… I've struggled to concentrate on some of the writing (012, 54y). |
| Top three reasons for declining:   1. No reason, 2. Too weak/frail 3. Did not want to engage with digital technology | Participant Challenges to Enrollment |  |  |  |
| Full PRO data sets were successfully collected for 12/18 participants | Data collection |  | Awareness | **P:**…I think it's [ExerciseGuide UK] very, very good. And I think the main thing that you and your colleagues have got to possibly get through to people is to try and get them interested… It's about making people aware that there is this you can do (005, 77y) |
| 25.35% reported no access to digital technology | Technology Engagement | Technology and Platform Usability | Digital Literacy | **P:** Working with a package like that, I think they [older adults] would find that bit daunting. Especially people my age, younger people maybe would find that easier (002, 75y) |
| Approx. 1/5th declined due to not wanting to use digital technologies |  |  |  |  |
| SUS Scores: Excellent: 2; Good: 6; Ok: 0; Poor: 2; Awful: 2. Overall score: 72%. |  |  |  | I can get round it better. But that can be said for most websites. You know practice makes perfect. But no. I found it usable. You know, even when you're a bit of a dinosaur like myself. I did find it usable easy enough. (017, 64y) |
|  |  |  | Usability and Navigation | funny enough, I wouldn't consider myself computer illiterate, but I found this very easy to use. I found no grave problems. And you know when it's set your lesson, you filled out, and then when it told you to "do this". You did that. It was easy to use. (005, 77y). |
|  |  |  | Digital and Exercise Self-Efficacy | I'm a technophobe. I'm a technophobe. So, I wrote it down [exercise plan] (013, 70y). |
|  |  |  |  | I am a little bit afraid that fact I've got osteoporosis as well, I won't say it has muddied the waters, as it hasn't, But it's a factor. You know, something you have to factor in yourself. On the results in that. Yes, it [ExerciseGuide UK] was specifically designed for lung cancer, and I realised that, but it's helped me overall, it has helped me overall. (017, 64y). |
|  |  |  |  | I'll be honest with you, because of the breathlessness. I, I was shying away from the cardio element because I wasn't comfortable. But now I found that I can get to a point where I can raise my heart rate for a prolonged period of time using the breathing. (001, 64y). |
| *Note: R: Researcher; P: Participant;* | | | | |
