# Supplementary material for: Feasibility and acceptability of ExerciseGuideUK for those living with and beyond lung cancer: a mixed methods study
Source: Support Care Cancer. 2026 Jun 12;34(7):646. doi: 10.1007/s00520-026-10858-w (PMC13260022; doi:10.1007/s00520-026-10858-w)
Supplement: Supplementary file 3 — Supplementary file3 (DOCX 42 kb) [file 520_2026_10858_MOESM3_ESM.docx]

Supplement 4: Pillar Integration Process Table discussing whether the ExerciseGuide UK platform is acceptable for those Living with and Beyond Lung Cancer.

| **Quantitative Findings** | **Quantitative Categories** | **Pillar Building Themes** | **Qualitative Categories** | **Qualitative Findings** |
| --- | --- | --- | --- | --- |
|  | |  |  | |
|  |  |  |  |  |
| The website was credible: 4.38 (± 0.65) | Credibility and Usability | Affective Attitude  (*How an individual feels about the intervention*) | Exercise Experience | **P:** I think it's just that enjoyment of doing it [exercises], and I do feel it has. I've noticed that. It is. It is improving some muscle in my legs which I lost quite a lot of. Yeah, I just find it enjoyable to do. (002, 75y) |
|  |  |  |  | **P:** I actually look forward to doing my exercises…Well, to be honest, all the time I've been in this, I've enjoyed, you know, doing the exercises. (013, 70y) |
|  |  |  |  | **P:** Yeah, I'm not particularly into exercise…I think if my wife wasn't still behind me, I don't think I would have done it. (008, 74y) |
| SUS Scores: Excellent: 2; Good: 6; Ok: 0; Poor: 2; Awful: 2. Overall score: 72%. |  |  | Emotional Engagement | R: How have you found the last few weeks using the ExerciseGuide? In terms of any thoughts that really come to mind.  P: I found absolutely fine. I have no problem doing it all. (009, 63y) |
|  |  |  |  | P: It didn't take a great deal of motivation or remembering. I just thought I've got to do that, and it was just straight on with the exercises. (017, 64y) |
|  |  |  |  | P: I think because it was the chance to do something positive. And something, you know, this [cancer] is something I'm not going to recover from. I think an exercise programme like this. I felt as if I was doing something positive. And this it's good for you mentally as well, isn't it?  R: Yes. Yeah. You experienced both of them physical and those mental benefits as well?  P: Yeah, yeah, because without. How I would have still continued with, you know. My normal activities but. But there was something missing still, and this [ExerciseGuide UK] has filled that really for me. (002, 75y) |
|  |  |  |  | R: What was it that got you into the study? What was your motivation?  P: Simply you [doctoral student] asking me to…you asking me because, you know, you can have leaflets and things like that. But some people wouldn't even bother reading them, would they? But yeah, it was you saying, 'would you mind getting involved' and my main concern was the computer work. But no. But that's why I got involved with it because you mentioned it today. You said for research. Would you be prepared to do it? And I said, 'OK, yeah'. (005, 77y) |
|  |  |  |  | P: I think it's [ExerciseGuide UK] very, very good. And I think the main thing that you and your colleagues have got to possibly get through to people is to try and get them interested… It's about making people aware that there is this you can do, you know, don't just sit on the couch all day, have a go at this. (005, 77y) |
|  |  |  |  | P:…I do think it's a pity that it couldn't be available to more people, but you know, some I know wouldn't be able to do it. (006, 62y) |
| The website was presented professionally, with appropriate content, colour, and images: 4.15 (± 0.69) | Presentation |  | Visualisation and Design | P: Well, me personally, I just thought. I thought it was set out well. I thought there was lots of explanatory things. (014, 71y) |
|  |  |  |  | Yes, I like them [the animations]. They were clear. They were quite clear. I like the colours of them. Yeah, I wouldn't change them at all. They were great. (012, 54y) |
|  |  |  |  | P: You know, because I'm a teacher, I'm always really aware of dyslexia and things like that. So yeah, I think it was really friendly colours. (003, 51y) |
|  |  |  |  | P: To be honest, I thought it [ExerciseGuide UK] was very good. I'll keep it on my phone. (001, 64y) |
| The website added additional burden on me personally: 1.62 (± 0.65) | Additional Burden | Burden  (*The perceived amount of effort that is required to participate in the intervention*) | Treatment Challenges | R:…was there any times where you didn't really want to use the website or exercise?  P: Yes. Particularly around three days after the chemo, you know? That really knocked me out for about three days…I just got knocked out. (008, 74y) |
|  |  |  |  | P: So, one of the side effects for me of the medication, you know, it's an effect of my eyesight. So, you know, reading things has became a bit of a chore… I've struggled to concentrate on some of the writing. (012, 54y) |
| The website was unnecessarily complex: 2.15 (± 1.14) |  |  | Digital Literacy and Perceived Complexity | P: Working with a package like that, I think they [older adults] would find that bit daunting. Especially people my age, younger people maybe would find that easier. (002, 75y) |
|  |  |  |  | P: Like a lot of older people, I don't think. Would rather work from a booklet with those exercises in. And they would find it easier and more likely to make it a routine. Because they would possibly find. Going into the exercise guide on a PC, say, may be a little bit daunting. (002, 75y) |
| The website evoked negative emotions: 1.54 (± 0.78) | Emotional Burden |  |  | P: I can get round it better. But that can be said for most websites. You know practice makes perfect. But no. I found it usable. You know, even when you're a bit of a dinosaur like myself. I did find it usable easy enough. (017, 64y) |
|  |  |  |  | P: We [participant and wife] found it quite easy to use it. I'm lucky to have someone close to me to ask if I need to. But I thought it was quite easy. (004, 84y) |
| The website content was easy to understand: 4.38 (± 0.65) | Understanding and Navigation |  | User Experience and Navigation Ease | P: …funny enough, I wouldn't consider myself computer illiterate, but I found this very easy to use. I found no grave problems. And you know when it's set your lesson, you filled out, and then when it told you to "do this". You did that. It was easy to use. (005, 77y) |
|  |  |  |  | P: Yes, I thought it was quite good because. What sort of thing you go on to, things like this, there's loads of colours and loads of things going on. I like the fact that with this there was only a couple of colours in there, you know, with the things that you really need to stand out. So, like things that underlined in the pink. So yeah, I found it very easy to use. (003, 51y) |
|  |  |  |  | P: Yes, yes. And the diagrams, yes.… Like I said, I've not found any difficulty with it at all. (014, 71y) |
| The website was easy to use and navigate: 4 (± 1.15) |  |  |  | R: In terms of the exercise, was the information clear enough?  P: Oh yes, that was very clear. Yes. I mean, to be honest, even while doing the exercises, I actually had the web page open. And went back to it to make sure I was doing it right, you know, that sort of thing. You know, like the arm stretch, just to see if I was doing it right. And yes, I saw was doing it right and there we go, question answered. (013, 70y) |
|  |  |  |  | R: Breathing exercises on the exercises as well. What are your thoughts on the instructions?  P: Oh, straightforward. straight forward. Yeah. (017, 64) |
|  |  |  | Questions | R: How did you feel about the number of questions we asked you?  P: I thought they were fair, you know, fair enough. There wasn't too much, and you did you did provide a progress bar for most of them, if not all of them. Which always does help. Because, you know, you think sometimes surveys are never-ending…On the whole, the phraseology of the questions, they were good. (017, 64y) |
|  |  |  |  | P:…It kinda went on about the same thing, you know, more than once. (011, 76y) |
|  |  |  |  | P:…I used to think to myself, haven't already answered this, but in a different, in a slightly different way. (012, 54y) |
|  |  |  |  | R: How were the questions?  P: They drove me mad at times…the amount. Yeah, the amount. (006, 62y) |
|  |  |  | Work Commitments and Time Management | P: I do really struggle with the motivation because normally, when I'm in the work situation. I work 12-hour days. So, you know, I'm absolutely exhausted. (003, 51y) |
|  |  |  |  | P: I don't know how I would fit it in when I go back to work. You know, it's not motivation. But the exercises, they don't take hours out of your day, and that's the nice bit about it. They literally take, you know, maybe 15 minutes. So, I probably would keep them up. (003, 51y) |
|  |  | Ethicality  (*The extent to which the intervention has a good fit with an individual’s value system*) | Alignment with Personal Values and Goals | P: I always knew how important exercise is. You know, having the attitude that is important. And this has made me, this is made me actually involve myself and exercise (003, 51y) |
|  |  |  |  | P: I can't imagine life without exercise. (009, 63y) |
|  |  |  |  | R: So, one of the things you mentioned there was before we kind of met was that you were getting frustrated with your lack of physical fitness.  P: Yes, I actually been on Google and things like that, you know, trying to find exercises I could do. But all they said was do walking, but all they bloody doing is walking. (013, 70y) |
|  |  |  |  | P: Even before you introduce me to the study, well into the study I had searched out various websites (017, 64y) |
|  |  |  |  | R: Did you find it easy to start activity programme?  P: Yes, yes, it was something I actually wanted to do. Because, you know, I was realising I was hitting brick walls, getting out and doing things and hopefully improves me, and I was right. I did want to do some exercise, but I couldn't do the exercises that I used to do. You know whether it be getting back to playing table tennis or not. (004, 84y) |
|  |  |  |  | P: I knew I was getting worse, and I knew I would definitely have gotten worse if I didn't do some exercise. I know the benefits. And I do want to get better. (004, 84y) |
|  |  |  |  | P: It [exercise] will be definitely something I carry on doing exercise until I physically, and I mean physically, can't do. (012, 54y) |
|  |  |  |  | P: I wanted to carry on being active. And not being overwhelmed with the diagnosis… (009, 63y) |
|  |  | Flexibility  (*The extent to which the intervention components can be used differently without changing the core effective ingredient*) | Adaptability of Exercise Materials | P: You know, [I] printed out the exercises. And the less than the days in the weeks, and I just followed that. To the letter, really. Which again has now got me into the routine were. I would find it difficult not to do the exercises. Because again, it's. It's a daily routine now, isn't it? (002, 75y) |
|  |  |  |  | R: What did you find the benefit of having a physical copy of this instead of having it on your laptop?  P: You know, having it just there in the spare room, just having it right next to me. (009, 63y) |
|  |  |  |  | P:…I think the vast majority of, of people, of older people would work just as well with a printed booklet of exercises. You know, tailored too certain. Depending on people's physical capabilities at the time. (002, 75y) |
|  |  |  | Preferences for Tracking | P: I like to keep it [tracking] on paper. Just to be fair, I like to do it on paper. (013, 70y) |
|  |  |  |  | R: So, you took a more paper-based approach, then?  P: Yes, yes, absolutely. It could be my age. I like things in paper. I like to see things written down.(004, 84y) |
|  |  |  |  | R: Was there any particular reason why you didn't use the online tracking module?  P: Erm, not really, no. I had it printed out and made notes, so I didn't bother going online to do it. (003, 51y) |
|  |  |  |  | P: And I thought you know what? Me and him talk anyway, so I'll just tell him how well I'm doing. But yeah, my phone and watch. Have been my personal way of tracking, I suppose. (012, 54y) |
| The website features were well integrated: 3.92 (± 0.64) | Integration of Features | Intervention Coherence  (*The extent to which the participant understands the intervention and how it works*) | Goal Setting | P: But I think if you don't have a goal, I mean, you want something to aim at. I want something to aim at so I can judge where I am. And if you don't have a goal, you will just tail it off... (004, 84y) |
|  |  |  |  | P: But I think if you don't have a goal, I mean, you want something to aim at. I want something to aim at so I can judge where I am. And if you don't have a goal, you will just tail it off. I don't like giving up. I don't like failure. You know, if you don't have a goal, you will just think, why bother? You know you can go down a slippery slope if you don't have a goal. (004, 84y) |
|  |  |  |  | P: But like I said at the beginning, I have found this training, you know, this exercise different from anything I've done before because one of the main things is you encouraged us to set our own targets. It made me feel more confident. Because I felt like I exceeded my own targets, and it makes you realise that you don't have to do a lot of exercise. You know, to achieve a good workout. (012, 54y) |
|  |  |  |  | P: I just followed that [exercise plan]. To the letter, really. Which again has now got me into the routine were. I would find it difficult not to do the exercises. Because again, it's. It's a daily routine now, isn't it? (002, 75y) |
|  |  |  |  | P: Once you've got it [exercise plan] into your head, you just go through them. Almost. You know, without thinking about it. And it becomes. More of a habit if you like. And I didn't mind that because it helped me to do it. (011, 76y) |
|  |  |  | Exercise Efficacy and Adaptation | P: I really did come to realise that every little does help. Every little is not little. Every little is something. And I've really came to realise that.  R: And when you said every little, what is it? What is it you referring to?  P: Exercise or just doing things in general. (017, 64y) |
|  |  |  |  | P: …also, I've got cancer, you know, can I exercise? You're asking yourself that question when you're starting. Should I be exercising? Should I be rocking in a corner somewhere? So, you think to yourself, why bother? Why bother? What's it going to do? I'm dying. What's it going to benefit me? It's not just about the physical stuff. It's your brain, isn't it? It's those little things that we talked about them achievements, that sense of belonging. Putting yourself little targets in and achieving them… (012, 54y) |
|  |  |  |  | R: So how did you find the exercises? You know, the progression, the information, how do you find all that?  P: Yeah, definitely good. Yeah, you know, it went up. They went up as the weeks went along, didn't it? You know, the amount of reps of the actual exercises went up, so I thought that was good. (003, 51y) |
|  |  |  |  | P: You know, I'm on week nine now. I'm hoping in a couple of weeks' time, instead of doing 12 repetitions, I'm hoping I can take up to 15. In that kind of things, and that's in the future, but I'm quite happy the way I am now. Things are going nicely for me. I'm doing the exercises fine now…(013, 70y) |
| The action planning tool/activity was useful to me: 4.08 (± 0.64) | Usefulness of Features |  | Website Architecture and Structure | P: I think I would have done the lot [the modules] and gone to the end. And not done the build-up. I would have just wanted to achieve, maybe. And I like the fact that just held me back a little bit at work, personally for me. (012, 54y) |
|  |  |  |  | P: Yeah, I think it's better releasing them [modules] over the course of the weeks because, you know, if you do more, like. If they were all there with the start, I would have done all them at the start. And then I probably would have forgot the stuff. So, you know, especially with chemo brain, it's nice that staggered. (003, 51y) |
|  |  |  |  | R: Do you think that staggered approach was useful?  P: I do I do because…you can be swamped by it, you know. I'm not really a reader…If you were given access to everything. It could put a lot of people off. So, the way it's done. It's in small chunks, it guides you through. (001, 64y) |
|  |  |  |  | R: What your thoughts on that sort of staggered release versus  P: I think. Leave it like that, because if you give them something all at once, they get quite overwhelmed. It's a lot to digest. You know they've got enough going on as it is. (009, 63y) |
| The resources Extra Information/Library was useful to me: 3.77 (± 1.09) |  |  |  | P: With people like me. Like the breathing, you know. Because with lung cancer, it's gonna affect your breathing. So that could come up fairly early and signposted to it little bit more. (001, 64y) |
|  |  |  |  | R: Was there any reason that it [Tracking module] wasn't used, or did you have another way of tracking offline?  R: Initially, it wasn't clear… (017, 64y) |
|  |  |  |  | R: There was a tracking module that you didn't really use. Was there any particular reason why you didn't engage with that module?  P: I don't really remember seeing it. (008, 74y) |
|  |  |  |  | R: And is that something that you think you would like to do or would have done?  P: Yes, yeah, I mean, knowing it's there is, this is the thing… (017, 64y) |
|  |  |  |  | R: So, you mentioned you didn't look at the actual information page. So, was there any particular reason for that?  P:…I don't think it was obvious. (008, 74y) |
|  |  |  |  | R: So, there was a bunch of extra information on the website that I don't think you accessed, was the reason for that?  P: I probably didn't see it. If I saw something, I went through and had a look at it, so I probably didn't see it. (005, 77y) |
| The website met my expectations: 3.85 (± 1.07) | Expectations | Perceived Effectiveness  (*The extent to which the intervention is perceived as likely to achieve its purpose*) | Habit Formation | P: Whereas now. I would find it difficult not to do it if that would make sense…It [exercising] becomes very much part of your day. And your daily routine. (002, 75y) |
| The website was interesting 4.23 (± 0.73) |  |  |  | P: I mean, today, for example, I was making a pie today, you know, I was in the kitchen, and in the kitchen is my two bottles of squash. And I use them for my arm exercises instead of the tins of beans used two bottles of squash. And while I was waiting, you know, doing little bits with the pie. I just noticed them, and I just. I think it just comes natural, I just grab hold of them and start doing, you know, a few bicep curls and this, that and the other. (013, 70y) |
| The website helped me to identify my physical activity goals: 4.38 (± 0.65) | Behaviour Change |  |  | R: Was there any impact on views regarding healthy lifestyle choices or diet?  P: No, not really. I don't really do a healthy lifestyle. (008, 74y) |
| The website is something I would like to continue to use on a regular basis: 3.54 (± 1.33) |  |  |  |  |
| The website has changed my attitude toward participating in physical activity throughout the cancer journey: 3.77 (± 0.93) |  |  |  | R: Do you think there's been any impact towards your attitude towards exercise?  P: I think when I started on week four and week five. I actually realised how much, how much this is starting to do for me. (011, 76y) |
| The website was relevant to me personally: 4.15 (± 0.99) | Personal |  | Tailoring, Personalisation, and Check-Ins | R: Did the website tailor the exercise is enough to you, do you think?  P: Yes, yeah, yeah. (014, 71y) |
| The modules were useful to me: 4.23 (± 0.73) |  |  |  | P: I liked the way that when you put your details in it, it gave you exercises, right, that were applicable to you, because the exercisesyou gave for my core stability and my hips. I've now incorporated that. And I will carry on having that in my workouts. (001, 64y) |
|  |  |  |  | R: What are your thoughts on the websites, tailoring ability and its ability to personalise it to your needs?  P: Yes, yes, I think it was very good. And I was amazed at the speed it did it with. You know. When we met as well when we spoke, you were very quick to change things which was great. (005, 77y) |
|  |  |  |  | P:…what the assessment does and what the, what the outcome of that assessment is, is basically what what the PT [personal trainer] first did for me when I walked into a gym. And that was huge. And I told him that all the problems I had. And they did draw me a plan up. (001, 64y) |
|  |  |  |  | R: I know we did talk about the website did tailor the exercises to you, but I [researcher] did do some extra tailoring.  P: Which is really helpful as you know what you're talking about. (013, 70y) |
|  |  |  |  | P. You know, when I first saw it. And I saw all the animations. You know, I thought that's not, that is really what I wanted is it's doing a whole body. You know my legs, my arms. I think I took a liking to it straight away, if you know what I mean. When I did the very first one, I thought oh, this isn't too bad. Well, I didn't say that. I actually thought this is good. I started off with some positive thinking. This will do me good as well. That was my first thoughts. When I very first did them, I was really pleased that someone came up with something tailored to me. (013, 70y) |
|  |  |  |  | P:…I think that was about right [number of check-ins]. I don't want to see you every week. But yeah, I think it's about right. (004, 84y) |
|  |  |  |  | P: I think it [number of check-ins], it's probably, it’s been enough for me. (011, 76y) |
|  |  |  |  | R: Would you have been less inclined [to do the exercises] if we didn't talk on the phone?  P: Only slightly [researchers name], only slightly, to be honest. But I would say talking with you it's motivated me a bit. (013, 70y) |
|  |  |  |  | R: Did meeting with me twice have any impact on your desire or willingness, or motivation to do exercise?  P: I think I can easily say yes. It had an impact on my motivation. (017, 64y) |
|  |  |  | Physical and Psychological Benefits | P: It [ExerciseGuide UK] boosted my confidence to no end, yeah. (013, 70y) |
|  |  |  |  | P: It was quite difficult to even raise my arms a little bit at the beginning. But now I'm doing that with weight. So definitely improvement in strength there. But not only that, the leg ones are really helped as well. (003, 51y) |
|  |  |  |  | P: I wince, and I do them [the exercises]. It's not very enjoyable throughout, but you know, the result, you know. Seeing the improvements is what I enjoy, and I have improved a bit. I think if I hadn't done the exercises, I wouldn't be where I am today. (004, 84y) |
|  |  |  |  | P: I've notice that these activities [activities of daily living] are a bit easier, so, you know, I've had trouble lifting things down from shelves before. And you know. Carrying things like water, but yeah, I've definitely, these are better. So now I just do these things, not even thinking about it, really. (003, 51y) |
|  |  |  |  | P: …there is a benefit, and it's the benefit you don't realise until the benefits there. For an example, bending. Bending to put your socks on. That used to be a bit of a problem for me. But now, after doing exercise, that isn't a problem. (005, 77y) |
|  |  |  |  | P: You know, I've just climbed them stairs. I didn't have to take a pause for breath. Mentally, that does you good. (004, 84y) |
|  |  |  |  | P: It's not just about the physical stuff. It's your brain, isn't it? It's those little things that we talked about them achievements, that sense of belonging. Putting yourself little targets in and achieving them. (012, 54y) |
|  |  |  |  | P: I think I always feel better mentally once I've exercised. You know, it does give you those endorphins, doesn't it? (003, 51y) |
|  |  |  | Managing Breathlessness | R: So, managing your breathing better has allowed you to do more activity.  P: Yes, yeah, yeah.(014, 71y) |
|  |  |  |  | R: So maybe that diagnosis introduced a barrier for your exercise.  P: It did. But I physically couldn't do it [exercise] because, you know, I couldn't breathe properly. So that was true. I couldn't do it [exercise]. But I can do it now! (006, 62y) |
|  |  |  |  | R: Did you have any barriers? To being active, to being physically active.  P: Mainly the breathing.  R: And has there been any changes with your breathing?  P: Yes, definitely. Definitely. Even with the walking about and things, you know, that's definitely helped me [breathing exercises]. (014, 71y) |
|  |  |  |  | P: One of the biggest things I have learned from it now, and it's part of me now, is that I'm not afraid of being breathless. Right. I know if I do something, or if I've overdone it or something, and I'm really breathless, I sit down, and I do my breathing exercises through diaphragm. And I'm relaxed. I know, I'm not gonna die. It's not gonna do anything. It's just going to go away in a couple minutes, and in a couple minutes I am back to normal. And that is the biggest, the biggest thing it taught me. I am not frightened of it. (013, 70y) |
| The website added (or would add if you are post treatment) value to my cancer care and service: 4.08 (± 0.86) | Value to Cancer Continuum |  | Value to Cancer Continuum | R: So, what's your thoughts about this [ExerciseGuide UK] being a part of someone's cancer journey?  P: Absolutely. Absolutely. (009, 63y) |
| The website could be easily integrated into a part of routine care for individuals receiving a lung cancer diagnosis: 4.23 (± 0.83) |  |  |  | P: I found them [the modules] interesting and beneficial. (005, 77y) |
|  |  |  |  | P: But yeah, I thought it [ExerciseGuide UK] was good. It's very helpful. It's a go-to place. (001, 64y) |
|  |  |  |  | P: …also, I've got cancer, you know, can I exercise? You're asking yourself that question when you're starting. Should I be exercising? Should I be rocking in a corner somewhere? So, you think to yourself, why bother? Why bother? What's it going to do? I'm dying. What's it going to benefit me? It's not just about the physical stuff. It's your brain, isn't it? It's those little things that we talked about them achievements, that sense of belonging. Putting yourself little targets in and achieving them… (012, 54y) |
|  |  |  |  | P: …other places you could go for advice I didn't always go to. Those places that was suggested, but [I] did go to some. And I did find some of them useful, some in particular, yeah. (017, 64y) |
|  |  | Self-Efficacy  (*The participants confidence that they can perform the behaviour(s) required to participate in the intervention*) | Digital Confidence | P: Yes, I don't think the tracker was as important. I like to write things down. I know, I like to write the exercise and tick them off when I've done them and if I need to write notes I will.  R: How do you feel about paper tracking log? Do you think that will be any use?  P: I think it will be. (005, 77y) |
|  |  |  |  | P: I'm a technophobe. I'm a technophobe. So, I wrote it down [exercise plan]. (013, 70y) |
| The website has increased my confidence to participate in physical activity: 4.08 (± 0.86) | Exercise Confidence |  | Exercise Confidence | P: I'll be honest with you, because of the breathlessness. I, I was shying away from the cardio element because I wasn't comfortable. But now I found that I can get to a point where I can raise my heart rate for a prolonged period of time using the breathing. (001, 64y) |
|  |  |  |  | P: I am a little bit afraid that fact I've got osteoporosis as well, I won't say it has muddied the waters, as it hasn't, But it's a factor. You know, something you have to factor in yourself. On the results in that. Yes, it [ExerciseGuide UK] was specifically designed for lung cancer, and I realised that, but it's helped me overall, it has helped me overall. (017, 64y) |
|  |  |  |  | R: Do you think this [exercise] is something that you would keep doing in the future?  P: yes, yeah, certainly. You know, maintaining the level I'm at certainly depending whether I want to go further. The scheme exercise plan] I have at the moment I'm very comfortable with, and I'll keep on going, you know, getting on the carpet and doing Superman. I'm quite happy to carry on doing that. As long as you know, I feel fit. You know. You know if it's doing good, which I think it has done good.. (004, 84y) |
|  |  |  |  | P:…the floor exercises, the crunches, and you know, the heel touches. When I first did them, I thought this is a big mistake. I'm going to cause myself some mischief because I can see my blood pressure going through the roof or something. But I persevered. But now I can just get on the floor and do them…I was struggling to get off the floor and using the stick. And now I can just stand up. (011, 76y) |
|  |  |  |  | P: I think satisfaction, yes, you know, knowing that you can still do these things [exercise] and knowing that help in your body. (003, 51y) |
|  |  |  |  | P: Because of the restrictions on me, on me, my exercises have to be done in the house, and I feel good about doing them. (017, 64y) |
| Notes: | | | | |
