# Supplementary material for: Feasibility and acceptability of ExerciseGuideUK for those living with and beyond lung cancer: a mixed methods study
Source: Support Care Cancer. 2026 Jun 12;34(7):646. doi: 10.1007/s00520-026-10858-w (PMC13260022; doi:10.1007/s00520-026-10858-w)
Supplement: Supplementary file 4 — Supplementary file4 (DOCX 25 kb) [file 520_2026_10858_MOESM4_ESM.docx]

Supplement 5: List of all modules and description, tailoring process, and mechanism of action for ExerciseGuide UK.

| **Module** | **Module Description** | **Tailoring** | **Mechanism of Action** | **Mapped to BCT^🞟^** | **Application in ExerciseGuide UK** |
| --- | --- | --- | --- | --- | --- |
| Getting Started | Introductory module to the website. Demonstrating how to use and navigate the website. | No tailoring | - Knowledge - Self-Efficacy | - Health consequences (BCT 5.1) | ExerciseGuide UK utilised videos and reinforcing messages to build Knowledge (MoA) and enhance Self-Efficacy (MoA), applying Information about health consequences (BCT 5.1) to demonstrate app usage and benefits |
| Physical Activity Programme | Provide a personally tailored physical activity programme in two sections. Section one will cover week one to week three. Section two will cover week four to week eight. Additionally, introductory safety information is provided. | Tailoring was based on pre-set questions which covered prior physical activity and exercise experience, physical health limitations. | - Knowledge - Self-Efficacy - Intentions | - Goal setting: behaviour (BCT 1.1) - Problem Solving (BCT 1.2) - Goal setting outcome (BCT 1.3) - Health consequences BCT 5.1) - Instruction on how to perform the behaviour (BCT 4.1) | Personalised exercise programmes were designed to increase Knowledge, Self-Efficacy, and Intentions (MoAs) through Goal setting (BCT 1.1), Action planning (BCT 1.2), and Instruction on how to perform the behaviour (BCT 4.1) |
| SMART Goals | Provide information regarding SMART goals. Linked to the Action Plan. Participants will set their own SMART goal. | Personalised introduction with messaged based on previous goal setting habits. | - Knowledge - Goals/Behavioural Regulation - Intentions - Motivation | - Goal setting: behaviour (BCT 1.1) - Problem Solving (BCT 1.2) - Goal setting outcome (BCT 1.3). | Educational content on SMART goals aimed to enhance Knowledge, Goals/Behavioural Regulation, and Motivations (MoAs) using Goal setting (BCTs 1.1, 1.3) and Problem solving (BCT 1.2) |
| Action Plan | Supported by the SMART goals module, the Action Plan guides participants to set a personally relevant and meaning plan to achieve their SMART Goal. | Not tailored. Participants are guided to set an Action Plan with specific questions. Ultimately setting a personalised action plan. | - Goals/Behavioural Regulation - Intentions - Motivation | - Goal setting: behaviour (BCT 1.1) - Action planning (BCT 1.4) - Self-monitoring of behaviour (BCT 2.3) - Feedback on behaviour (BCT 2.2) - Intrinsic motivation (BCT 10.3) | Interactive SMART goal setting enhanced Knowledge and Motivations (MoAs) via Goal setting (BCT 1.1) and Adding objects to the environment (BCT 12.5) for tangible action planning |
| Exercise Safety | Provide safety information for those LWBLC regarding being physically active and engaging in exercise. | Further in-depth guidance is provided for specific health- and cancer-related concerns. | - Knowledge - Self-Efficacy - Beliefs about Capabilities - Needs - Perceived Susceptibility/Vulnerability | - Health consequences (BCT 5.1) - Persuasion to boost self-efficacy (BCT 15.1) - Problem solving (BCT 1.2) | Safety tips increased Knowledge and Self-Efficacy (MoAs) through Information about health consequences (BCT 5.1) and self-efficacy (BCT 15.3) for safe exercise practices |
| Exercise Benefits | Provide informative content surrounding benefits of physical activity for those LWBLC. | Health issues and cancer-related side effects which may be improved via physical activity and exercise. | - Knowledge - Optimism - Self-Efficacy - Intentions - Motivation - Beliefs about Consequences | - Health consequences (BCT 5.1) - Encouraging about past success (BCT 15.2) - Intrinsic motivation (BCT 10.3) - Material reward (BCT 10.2) - Information about emotional consequences (BCT 5.6) - Information about health consequences (BCT 5.1) - Information about social and environmental consequences (BCT 5.3) | Module to discuss exercise benefits aimed to enhance Knowledge, Optimism, and Self-Efficacy (MoAs) via Information about health consequences (BCT 5.1) and Information about emotional consequences (BCT 5.6). |
| Motivation | Content surrounding motivation, barriers and enablers to physical activity, and habit formation. | Identify and assistive feedback on specific barriers to physical activity and exercise. | - Emotion - Attitude towards the Behaviour - Values - Motivation - Automaticity | - Regulation of emotions (BCT 11.2), - Stress management (BCT 11.3) - Pros and cons (BCT 9.1) - Values clarification (BCT 13.1) - Prompts and cues (BCT 7.1) - Intrinsic motivation (BCT 10.3) | Motivational strategies addressed Emotion and Attitude towards the behaviour (MoAs) through Reducing negative emotions (BCT 11.2) and Identification of self as a role model (BCT 13.1). |
| Tracking Module | Provides an opportunity for self-monitoring of exercise and healthy lifestyle behaviours and outcomes. | No tailoring | - General Attitudes/Beliefs - Self-regulation | - Persuasive communication (BCT 5.4) - Information about emotional consequences (BCT 5.6) - Self-monitoring of behaviour (BCT 2.3) - Feedback on behaviour (BCT 2.2) | Weekly tracking fostered Self-Regulation (MoA) using Feedback on behaviour (BCT 2.2) and Self-monitoring of behaviour (BCT 2.3) via visual progress graphs. |
| Other Activities | Covers information regarding what is physical activity, exercise, and physical fitness. Further information regarding non-conventional activities and exercises. | Tailored information provided around methods of getting in ‘other’ types of activities within their daily lives. | - Knowledge - Optimism - Self-Efficacy - Intentions | - Instruction on how to perform the behaviour (BCT 4.1) - Health consequences (BCT 5.1) - Reduce negative emotions (BCT 11.2) | Expanding exercise understanding increased Knowledge and Self-Efficacy (MoAs) with Instruction on how to perform behaviour (BCT 4.1) and Reducing negative emotions (BCT 11.2) |
| Health Lifestyles | Provide informative content, both generally and lung cancer specific regarding lifestyle factors which may increase health-related quality of life. | Tailored information based on treatment type and lifestyle habits (smoking, alcohol, sleep, activity minutes), and personal values | - Knowledge - Optimism - Self-Efficacy - Intentions - Motivation - Beliefs about Consequences | - Instruction on how to perform the behaviour (BCT 4.1) - Health consequences (BCT 5.1) - Information about emotional consequences (BCT 5.6) | Lifestyle advice was provided to boost Knowledge, Optimism, and Self-Efficacy (MoAs) using Information about health consequences (BCT 5.1) and Emotional consequences (BCT 5.6) |
| Breathlessness | Provide foundational information of what is breathlessness, causes, and exercises to help (both video and written demonstrations). | No tailoring | - Knowledge - Self-Efficacy | - Instruction on how to perform the behaviour (BCT 4.1) - Health consequences (BCT 5.1) - Information about emotional consequences (BCT 5.6) | Information on breathlessness aimed to increase Knowledge and Self-Efficacy (MoAs) through Instruction on how to perform the behaviour (BCT 4.1) and Information about health consequences (BCT 5.1) |
| Mental Health | Provides an introduction to mental health and lung cancer. Additionally, this module provides multiple links to external sources which discuss lung cancer and mental health related factors. | No tailoring | - Knowledge - Signage and Support | - Social support (unspecified) (BCT 3.1) - Social support (practical) (BCT 3.2) - Prompts/cues (BCT 7.1) - Reduce negative emotions (BCT 11.2) | Mental health resources were linked to Knowledge and Support (MoAs) via Social support (unspecified) (BCT 3.1) and Prompts/cues (BCT 7.1) to encourage engagement with supportive materials |
| Note: LWBLC: Living with and beyond lung cancer; SMART: Specific, Measurable, Attainable, Realistic, Time-Bound.  **^🞟^**BCT; Behaviour Change Techniques taken from the The Behavior Change Technique Taxonomy (v1) of 93 Hierarchically Clustered Techniques ([459](#_ENREF_459)) | | | | | |
