# Supplementary material for: Feasibility and acceptability of ExerciseGuideUK for those living with and beyond lung cancer: a mixed methods study
Source: Support Care Cancer. 2026 Jun 12;34(7):646. doi: 10.1007/s00520-026-10858-w (PMC13260022; doi:10.1007/s00520-026-10858-w)
Supplement: Supplementary file 5 — Supplementary file5 (DOCX 29 kb) [file 520_2026_10858_MOESM5_ESM.docx]

Supplement 6

*Supplement 6.1: Patient-reported outcome measures over the eight-week ExerciseGuide UK intervention*

|  |  |  |  | Bootstrap^a^ | | |  |
| --- | --- | --- | --- | --- | --- | --- | --- |
|  | Baseline  (n=12) | Post  (n=12) | Mean Change (SDC) | P value | CI (95%) | |  |
| EORTC QLQ-C30 | | | | | | |  |
| Quality of Life^1^ | 45.14 ± 24.24 | 50 ± 32.37 | 4.86 (27.86) | 0.554 | -10.42 - 20.14 | |  |
| Functional Scales^2^ | | | | | |  | |
| Physical Functioning | 77.78 ± 23.67 | 81.11 ± 18.61 | 3.33 (22.30) | 0.618 | -8.33 - 15.56 |  | |
| Role Functioning | 76.39 ± 29.70 | 75 ± 27.98 | -1.39 (29.70) | 0.88 ^b^ | -18.06^b^ - 13.89 ^b^ |  | |
| Emotional Functioning | 81.95 ±23.79 | 83.33 ± 23.00 | 1.39 (9.67) | 0.906 | -17.36 - 19.44 |  | |
| Cognitive Functioning | 83.33 ± 17.41 | 76.39 ± 27.94 | -6.94 (9.28) | 0.490 | -26.39 - 9.72 |  | |
| Social Functioning | 76.39 ± 29.69 | 73.61 ± 29.69 | -2.78 (47.05) | 0.852 | -27.78 - 23.61 |  | |
| Symptoms Scales^3^ | | | | | |  | |
| Fatigue | 34.26 ± 34.31 | 27.78 ± 30.15 | -6.48 (40.33) | 0.603 | -29.63 - 14.81 |  | |
| Nausea and vomiting | 9.72 ± 19.41 | 11.11 ± 21.71 | 1.39 (31.35) | 0.888­­^c^ | -15.28^c -^ 19.44^c^ |  | |
| Pain | 22.22 ± 29.59 | 06.94 ± 13.22 | -15.28 (32.14) | 0.178 | -34.72 - 0.00 |  | |
| Dyspnoea | 27.78 ± 34.33 | 6.94 ± 13.22 | -20.83 (35.62) | 0.109^d^ | -41.67^d^ - 5.56^d^ |  | |
| Insomnia | 16.67 ± 22.48 | 33.33 ± 37.61 | 16.68 (30.15) | 0.115^e^ | 2.77^e^ - 36.11^e^ |  | |
| Appetite Loss | 25.00 ± 40.51 | 16.67 ± 30.15 | -8.33 (53.42) | 0.598^f^ | -36.11^f^ -19.44^f^ |  | |
| Constipation | 25.00 ± 35.18 | 11.11 ± 25.95 | -13.89 (41.34 | 0.284^g^ | 36.11^g^ - 8.33^g^ |  | |
| Diarrhoea | 13.89 ± 30.01 | 11.11 ± 25.95 | -2.78 (43.72) | 0.842^f^ | -27.78^f^ - 19.44^f^ |  | |
| Financial Difficulties | 11.11 ± 21.72 | 8.33 ± 28.87 | -2.78 (38.82) | 0.821^h^ | -22.22^h^ - 19.44^h^ |  | |
| Hospital Anxiety Depression Scale (HADS)^4^ | | | | | | |  |
| Anxiety | 5.59 ± 5.14 | 4.50 ± 2.58 | -1.08 (3.55) | 0.319 | -3.08 – 0.75 |  | |
| Depression | 3.59 ± 2.81 | 3.17 ± 1.90 | -0.42 (0.45) | 0.394 | -1.33 – 0.42 |  | |
| Baseline and Post data is given in Mean ± Standard Deviation; Mean Change is given with Standard Deviation Change (SDC); CI = Confidence Interval; CI – 95%; CI given in Lower-Upper values; Analysis was conducted using a Post – Pre model. a - based on 10,000 samples, unless otherwise stated; b - based on 9998 samples; c - based on 9978 samples; d - based on 9936 samples; e - based on 9933 samples; f - based on 9989 samples; g - based on 9986 samples; h - based on 9900 samples. 1 – Quality of Life is scored on a scale of 0 – 100, with higher scores representing a higher quality of life; 2 – Functional scores are measured using a 0 – 100 scale, with higher scores representing a higher level of functioning; 3 – Symptoms scores are measured using a 0 – 100 scale, with higher scores representing a higher symptom burden/greater impact of symptom; 4 – Hospital Anxiety Depression Scale, which provided two subscales to measure anxiety and depression independently. A score higher than seven signifies anxiety or depression. | | | | | | | |

*Supplement 6.2: The Community Health Activities Model Program for Seniors (CHAMPS) Data*

|  |  |  |  | Bootstrap^a^ | |
| --- | --- | --- | --- | --- | --- |
|  | Baseline  (n=11) | Post  (n=11) | Mean Change (SDC) | P value | CI (95%) |
| Frequency (per/week) | 17.09 ± 10.17 | 25 ± 12.39 | 7.91 (15.51) | 0.132 | -0.64 – 16.82 |
| Frequency of Moderate per/week | 6.91 ± 7.09 | 8.73 ± 4.84 | 1.82 (2.35) | 0.468 | -2.73 – 6.45 |
| Duration (hours/week) | 10.25 ± 6.00 | 17.27 ± 9.28 | 7.02 (3.14) | 0.064 | 0.98 – 13.30 |
| Duration of Moderate (hours/week) | 4.27 ± 4.22 | 6.68 ± 3.89 | 2.41 (1.42) | 0.141 | -0.45 – 5.16 |
| Baseline and Post data is given in Mean ± Standard Deviation; Mean Change is given with Standard Deviation Change (SDC); CI = Confidence Interval; CI – 95%; CI given in Lower-Upper values; Analysis was conducted using a Post – Pre model. a: Unless otherwise noted, bootstrap results are based on 10,000 bootstrap samples. | | | | | |

**Bootstrapping Rationale**

Given the small sample size and violation of normality assumptions in the PRO data, a parametric paired samples t-test with bootstrapping (~10,000 resamples) was employed to explore mean change scores between baseline and follow-up. A parametric approach was preferred over non-parametric alternatives, such as the Wilcoxon signed-rank test, as reporting mean change provides a more clinically meaningful signal of effect than rank-based comparisons, particularly important in a small feasibility sample where statistical significance is not the primary aim. Furthermore, non-parametric tests exclude data ties, which are common in Likert-scale PRO measures such as the EORTC-QLQ-C30 and HADS, and would have further reduced an already small completer sample. Bootstrapping was therefore selected as it does not assume normality, retains all available data, and provides more stable confidence interval estimates from small samples [1, 2].

It is acknowledged, however, that with a completer sample of n=12, bootstrapped confidence intervals remain limited in their precision, as resamples are drawn from the original sample; findings should therefore be interpreted as exploratory signals of effect rather than definitive estimates, and population-based inferences cannot be made.

**Supplement 6 References:**

1. Efron, B. and R. Tibshirani, *The Bootstrap Method for Assessing Statistical Accuracy.* Behaviormetrika, 1985. **12**(17): p. 1-35.

2. Efron, B. and R.J. Tibshirani, *An introduction to the bootstrap*. 1994: CRC press.
