# Supplementary figures and images for: Feasibility and acceptability of ExerciseGuideUK for those living with and beyond lung cancer: a mixed methods study
Source: Support Care Cancer. 2026 Jun 12;34(7):646. doi: 10.1007/s00520-026-10858-w (PMC13260022; doi:10.1007/s00520-026-10858-w)

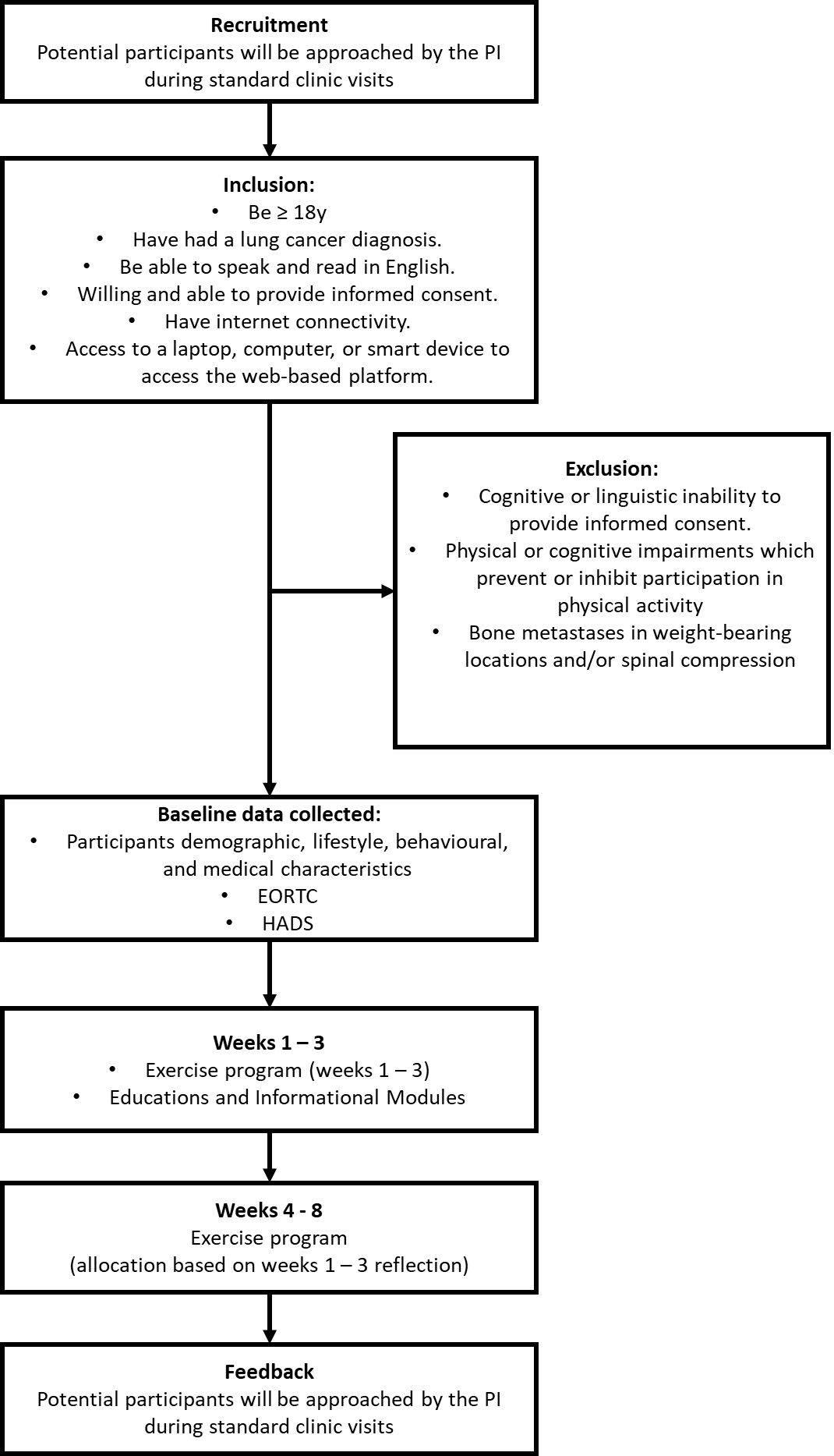


Supplement 1: Flow of participants through the ExerciseGuide UK study [16]

Supplement: Supplementary file 6 — Supplementary file6 (DOCX 70 kb) [file 520_2026_10858_MOESM6_ESM.docx]
